# ITRUSST Consensus on Standardised Reporting for Transcranial Ultrasound Stimulation

**Published:** 2024-02-15

**Authors:** Eleanor Martin, Jean-François Aubry, Mark Schafer, Lennart Verhagen, Bradley Treeby, Kim Butts Pauly

**Affiliations:** 1 Department of Medical Physics and Biomedical Engineering, University College London, London, U.K; 2 Wellcome/EPSRC Centre for Interventional and Surgical Sciences, University College London, London, UK; 3 Physics for Medicine Paris, Inserm U1273, ESPCI Paris, CNRS UMR8063, PSL University, Paris, France; 4 School of Biomedical Engineering, Science and Health Systems, Drexel University, Philadelphia, PA USA; 5 Donders Institute for Brain, Cognition and Behaviour, Radboud University, 6525 GD Nijmegen, the Netherlands; 6 Department of Radiology, Stanford University, Stanford, CA, USA

## Abstract

As transcranial ultrasound stimulation (TUS) advances as a precise, non-invasive neuromodulatory method, there is a need for consistent reporting standards to enable comparison and reproducibility across studies. To this end, the International Transcranial Ultrasonic Stimulation Safety and Standards Consortium (ITRUSST) formed a subcommittee of experts across several domains to review and suggest standardised reporting parameters for low intensity TUS, resulting in the guide presented here. The scope of the guide is limited to reporting the ultrasound aspects of a study. The guide and supplementary material provide a simple checklist covering the reporting of: (1) the transducer and drive system, (2) the drive system settings, (3) the free field acoustic parameters, (4) the pulse timing parameters, (5) *in situ* estimates of exposure parameters in the brain, and (6) intensity parameters. Detailed explanations for each of the parameters, including discussions on assumptions, measurements, and calculations, are also provided.

## 1 Introduction

Transcranial ultrasound stimulation (TUS) is a non-invasive neuromodulation technique that employs focused ultrasound waves to modulate neuronal activity within the brain. TUS offers a promising avenue for therapeutic and research applications due to its spatial precision and ability to target deep neural structures. As the field transitions to more widespread human studies, the time is upon us to standardize the reporting of such studies to aid understanding and reproducibility.

To this end, the International Transcranial Ultrasonic Stimulation Safety and Standards Consortium (ITRUSST) formed a subcommittee to review and suggest standardised reporting parameters for low intensity TUS, with multiple presentations to the ITRUSST group and multiple opportunities for feedback, resulting in the guide presented here. Similar guidelines have been produced for other analogous techniques and applications [1, 2].

This guide focuses on the ultrasound aspects of TUS experiments. It does not discuss reporting of additional elements such as EEG, fMRI, or behavioral readouts, which are important, but outside the scope of this work. It does not seek to establish safety values or thresholds, which are addressed in a separate ITRUSST consensus on TUS safety [3]. While this guide endeavors to align with existing international standards related to diagnostic ultrasound imaging and focused ultrasound transducers, it may diverge from these standards when necessary. An important additional note is that we make the assumption that the ultrasound fields that this guide applies to are linear. Our rationale for doing so is provided in Sec. A of the Supplementary Material.

This guide is structured as follows. In Section 2, we discuss reporting of the transducer and drive system, and the free field acoustic pressure amplitude and spatial characteristics of the focal region, as measured by a hydrophone in free field in a water bath. These measurements, performed under standardised conditions, serve as a baseline for comparison between studies independent from the specific configuration of use during TUS studies. In Section 3, we discuss the pulse timing parameters. Next, in Section 4, we discuss the reporting of exposure parameters, which describe the acoustic field inside the individual brain, after accounting for the skull bone, brain tissue, and any other acoustic distortions. Further details and a summary checklist of all required reporting parameters are provided in the Supplementary Material, Sections A–F.

## 2 System and Free Field Acoustic Pressure Parameters

System parameters describe the type and geometry of the transducer, and the signal chain or system used to drive the transducer in order to generate the acoustic field. The free field acoustic pressure parameters provide a description of the generated ultrasound field under reference conditions. The term ‘free field’ refers to the field generated when a transducer is radiating continuously [4] (or with the comparatively long pulses used in TUS studies) into water without any obstruction by reflectors, scatterers, or aberrators. See Sec. B for details of how this is implemented during measurements.

Reporting free field acoustic parameters allows comparison of the transducer output at the chosen output level and focal settings under standardised conditions, serving as a baseline for comparison between studies. In this guide, the term output level refers to the amplitude of the acoustic pressure output, which is governed by settings on the drive system such as voltage, power or intensity. The focal position setting refers to the position of the focal region where this can be steered to different positions. It may be fixed for a particular transducer, or set by the operator, for example, by selecting a distance setting, or by setting the relative phases applied to the transducer elements. The free field parameters described in this section should be reported at the output and focal settings used during exposure of participants during studies.

### 2.1 Transducer and Drive System

#### 2.1.1 Transducer Description

**Figure 1:**
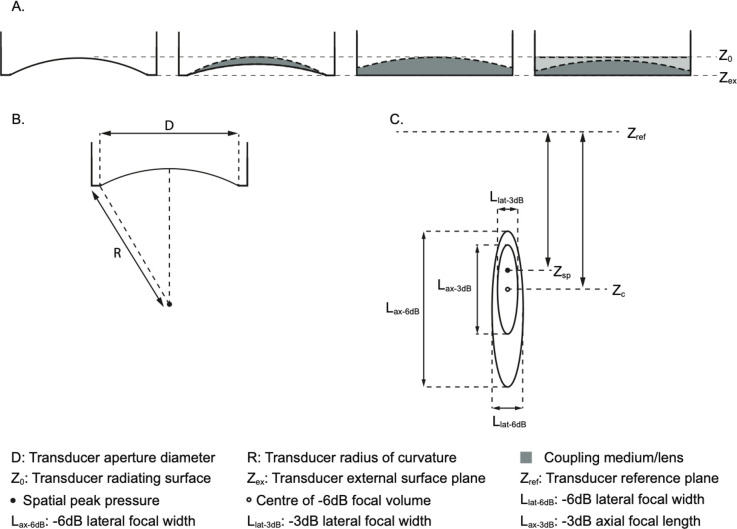
Schematics of example transducers, geometric and field parameters, for information. (A) common transducer configurations including attached coupling media or internal lenses. The external transducer surface plane, $Z_{ex}$ which may be coincident with the front surface of the transducer housing often serves as the reference plane, $Z_{ref}$ from which distances are measured. For some transducers, e.g. complex multi-element devices, $Z_{ref}$ may intersect with the centre of the surface on which the elements sit. (B) Aperture diameter, D, and radius of curvature, R, are used to describe the geometry of focusing transducers. (C) Free field pressure parameters shown with respect to the reference plane. $Z_{sp}$ is the axial position of the location of spatial-peak pressure relative to the reference plane $Z_{ref};\;Z_{c}$ is the axial position of the centre of the −3dB focal region relative to the reference plane.

A description of the ultrasound transducer should be given, including the manufacturer and model number, and the operating frequency of the transducer. For spherically focussing transducers, the geometry is described by the radius of curvature and aperture diameter (see Fig. 1). In some cases, the exact geometry may not be known, for example, if the transducer construction includes a lens or permanently attached coupling medium, in which case, any other information such as the nominal position of the focus relative to the transducer face should be reported. For multi-element transducers, the number of elements and information about their shape, size, and positions should be given if available. The exact size and position of elements may not be available for commercial transducers, so a brief description accompanied by the model number is sufficient. Any other features of the transducer such as permanently attached coupling media, or lenses should be described, including a description of the material, its thickness and shape/geometry, and properties of the material such as the sound speed, density and attenuation coefficient where known.

#### 2.1.2 Drive System Description

The input signal used to drive the transducer may be generated by an integrated drive system, or using a signal generator and RF amplifier. All components of the system, including manufacturer and model numbers and any external electrical impedance matching networks used to couple the drive signal to the transducer should be reported.

#### 2.1.3 Drive System Settings

All drive system settings used during studies should be described, including the operating frequency, output level settings (e.g. displayed electrical power, focal intensity, voltage etc.), and for multielement transducers, focal distance or position settings applied. The method of coupling the transducer to the participant forms part of the *in situ* acoustic transmission path, so the materials and methods used should be described.

### 2.2 Free Field Acoustic Parameters

#### 2.2.1 Parameters To Report

The free field acoustic pressure field parameters are illustrated in Fig. 1. Table 1.1 shows the quantities to be reported which includes spatial peak pressure amplitude, focal dimensions, and location. An explanation of how to calculate focal dimensions from the axial pressure profile is given in Sec. B.8. Two illustrative reporting examples are provided in Table 1.1, and annotated example axial pressure profiles are shown in Fig. B.2.

The amplitude of the spatial-peak acoustic pressure should be reported at each of the output level settings, and all quantities should be reported for each of the focal position settings used during studies, where practical. This will provide a reference spatial-peak pressure in water for each study exposure. For multi-element array devices where steering and aberration correction are performed, the focal position and amplitude may vary between participants and target locations. Therefore, reporting of free field parameters for each condition may not be useful. Instead information about the pressure amplitude and size of the focal region over the focal steering range utilised during the study should be reported. See Sec. C for recommendations for reporting average or ranges of parameters.

#### 2.2.2 Methods of Obtaining Free Field Pressure Parameters

The free field pressure parameters can be obtained by investigators in one of several ways, for example, directly from acoustic field measurements using a hydrophone in a water bath, from a test report provided by the manufacturer or other calibration/characterisation provider, or via a combination of simulation and measurement. In any case, the source and/or method used to obtain the field parameters should be reported, and where measurement or simulation were used, the procedures should be described, as well as the equipment used (including hydrophone type and element size, and calibration parameters). Further guidance can be found in Sec. B.

For those performing their own measurements to determine the free field acoustic pressure parameters, it is recommended that information on measurement best practice be sought from hydrophone measurement standards [7, 8, 4] with additional help from the literature (e.g. [9, 10, 11]). While a full description is out of scope of this document, some further details and discussion are provided in Sec. B. Similarly, where simulation is used to obtain some or all of the field parameters, the literature should be consulted for methods and best practice.

#### 2.2.3 Spatial-Peak Pressure at Study Output Levels

The free field spatial-peak pressure amplitude at each of the study output level settings and focal position settings may be obtained either directly from a measurement with the transducer operated at the study output level and focal setting, or by scaling a measurement made with the same focal setting, but at a different output level. Typically, a lower output level can be used for free field measurements than would be used to obtain a similar pressure amplitude in the brain after propagation through the skull. Measurements with sufficient signal to noise ratio can be made, while reducing the possibility of causing damage to the hydrophone (especially at the lowest frequencies). This is discussed in more detail in Sec. B.

#### 2.2.4 Defining the Spatial Location of Reported Free Field Pressure Parameters

The spatial location of the spatial-peak pressure amplitude can be defined relative to a variety of different reference locations, for example the radiating surface or the external transducer surface plane, also known as the exit plane (see Fig. 1). In some cases the full construction of the transducer or the distance from the radiating surface to the outer surface of any permanently attached or integrated lens or coupling medium may not be known. In this case, a useful reference location would be the outer surface of these layers, the external transducer surface plane. The chosen reference plane location should be reported along with the parameters.

#### 2.2.5 Uncertainty on Free-Field Acoustic Parameters

All hydrophone measurements have an associated measurement uncertainty which arises from a combination of systematic and random uncertainties. One major source of systematic uncertainty is hydrophone sensitivity, which can be up to 20% depending on frequency, and calibration method. Additionally, uncertainty on pressure measurements will propagate to derived quantities. Understanding of uncertainty on pressure measurements is essential when reporting and comparing measurements. Further discussion of this can be found in Sec B.3.

### 3 Pulse Timing Parameters

**Figure 2.**
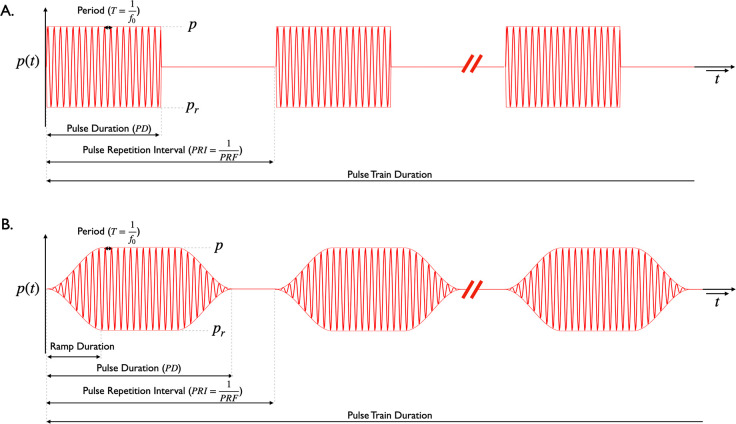
Schematics of two ultrasound pressure waveforms. A rectangular ramp shape is used in A, while a Tukey window on the pressure waveform is used in B. The pressure amplitude is shown as p, while the peak negative pressure is shown as $p_{r}$; these should be the same when operating at low pressures within the linear regime. The duration of a single cycle of the operating frequency is the period (T). A single continuous sonication is a pulse with a duration of pulse duration (PD). Pulses are often repeated in a pulse train. The time between two pulses in a pulse train is the pulse repetition interval (PRI) and is equal to 1 divided by the pulse repetition frequency (PRF). The pulse train has a duration which is the pulse train duration. The pulse train can be repeated, and if so has a structure similar to the pulse, as laid out in Tables 2.1–2.3. In this figure, the operating frequency $f_{0}$ is lower than typically used in order to aid visualization.

Typical pulse timing parameters are laid out in Figure 2.The period refers to the duration of one cycle of the operating frequency $f_{0}$. A pulse is a single continuous sonication and has a duration referred to as the pulse duration (PD). If the pulse is repeated, the pulse repetition interval (PRI) is the time between successive pulses. The pulse repetition frequency is given by $PRF=\frac{1}{PRI}$.

In Tables 2.1–2.3 we suggest a table reporting structure. In the rare case of only a single continuous pulse, only the first line of a table need be provided, with the pulse duration, and if a ramp is used, the pulse ramp duration and the pulse ramp shape. The pulse repetition interval is not applicable. When the pulse is repeated, the PRI/PRF are entered in the first line, and the second line provides information on the pulse train including the pulse train duration, the pulse train ramp duration, and the pulse train ramp shape.

The examples chosen from the literature illustrate a number of variations in TUS pulse parameters in order to illustrate increasing levels of patterning, as well as rectangular and ramped waveforms. While figures are helpful and illustrative, we feel that tables of the pulse parameters would be most clear.

A number of phrases are commonly used to refer to ultrasound pulse trains. We recommend avoiding ambiguous phrases while retaining clear conventions of the community. The use of the word “burst” is ambiguous as it has different meanings in different contexts. For example, in diagnostic ultrasound, burst refers to a single long pulse, while on function generators and in TMS, burst is often used to refer to pulse trains. We suggest avoiding the use of the word “burst” to remove this ambiguity and instead to use the terms “pulse” and “pulse trains.”

The phrase “repetitive TUS” (rTUS) is conventionally used to refer to pulse trains intended to elicit cumulative or delayed effects, as opposed to only acute effects. The distinction between acute and delayed effects is critical for study design (‘online’ vs. ‘offline’ designs), the underlying neurophysiology (acute modulatory vs. early-phase plasticity mechanisms), and the safety assessment. Distinguishing characteristics of an rTUS protocol, for example, the pulse repetition frequency, can be included in the label, as in the phrase ‘10 Hz rTUS’. It is recommended to avoid using unique labels when an (extended) rTUS label is also appropriate. By definition, all rTUS protocols include at least the pulse and pulse train timing parameters, as in the 5 Hz rTUS example A of Table 2.2. Patterned rTUS protocols are a subset of rTUS where the pulse trains are repeated in a pattern and, thus, include more than two rows, as in the patterned rTUS example B of Table 2.2.

For rectangular pulses in a single pulse train, duty cycle (DC) is defined as the percentage of time that a pulse is on,

(1) $$DC=\frac{PD}{PRI}100\%.$$

Duty cycle can be optionally reported since it can be easily derived from the timing tables and is only defined for rectangular pulses. However, if provided, we recommend referring to it in a particular way. The rTUS stimulation protocol of Table 2.2A has a single duty cycle of 10%. In this case, the overall DC is equal to the ${DC}_{pulse\;train}$. However, when rTUS is patterned, such as in the stimulation of Table 2.2B, it would be clearer to refer to the duty cycle of each level of patterning, such as ${DC}_{pulse\;train}$ and ${DC}_{pulse\;train\;repeat}$. The overall *DC* is then the product of each *DC*. For the patterned rTUS stimulation in Table 2.2B, ${DC}_{pulse\;train}=32\%,\;{DC}_{pulse\;train\;repeat}=31.25\%$, and the product is the overall duty cycle, DC=10%*.*

Note, our definition of the pulse duration differs slightly from the diagnostic ultrasound definition, which defines it as “1.25 times the interval between the time when the time integral of the square of the instantaneous acoustic pressure reaches 10% and 90% of its final value” [7]. This difference is negligible for long pulses.

While the community is only starting to embrace ramping as a means to reduce the auditory confound [5, 15, 16], it is likely that this will continue and potentially become a source of confusion unless reported in a standardised way. The shape and duration of the ramp must be reported as already described, as well as whether that shape is applied to the pressure (voltage) waveform or the intensity (power) waveform. In Table 2.1, we specify how ramping is applied in Johnstone *et al.* [5].

It is likely that the field will continue to evolve in ways that we cannot now anticipate. The table structure allows for evolution by adding columns or rows as needed.

## 4 Derived Parameters: *In Situ* Estimates of Exposure Parameters

Exposure parameters describe the properties of the acoustic field inside the individual brain, after accounting for the skull bone, brain tissue, and any other acoustic distortions. The exact ultrasound exposure is generally difficult to directly measure *in vivo*. Instead, exposure parameters are typically estimated using knowledge of the free field parameters along with a derating procedure, simulations, or in-direct measurements. Here, *in situ* estimate is used as a general term to describe the procedure used to calculate the exposure parameters, i.e., the *in vivo* or *in situ* properties of the acoustic field. The recommended *in situ* parameters are provided here, with examples provided in Sec D.

### 4.1 Estimated *In Situ* Pressure Amplitude

The skull bone has very different acoustic material properties (sound speed, density, and attenuation) to the surrounding soft tissues. As ultrasound traverses the skull, these differences cause the waves to become distorted and lose energy. Brain tissue also has a higher attenuation coefficient compared to water. Consequently, the acoustic focus inside the brain will generally have a much lower intensity (sometimes by a factor of 10 [17]). In some cases, the shape and position of the focus can also become distorted. To complicate matters, the shape and properties of the skull vary significantly both within and between subjects. The same system and pulse timing parameters can therefore generate very different acoustic fields in the brain depending on the subject and the position of the transducer. An estimate of *in situ* pressure amplitude should thus be reported. Several different approaches for obtaining this are outlined below. In all cases, the method used should be reported, along with details of any calculations, parameters, and assumptions.

- **Simulating the intracranial ultrasound field.** Simulations are increasingly used to calculate the ultrasound and temperature fields inside the skull and brain. The material properties for the simulations might be based on individualised images for each subject or a CT or MR template image.
  If using simulations to calculate the exposure parameters, the following should be reported:
  - Details of the simulation tool.
  - Details of how the acoustic and thermal material properties are assigned.
  - How the transducer modelling and positioning was performed within the simulation.
  - Details of relevant simulation input parameters, program settings including mesh or grid parameters, and processing steps.
  An excellent reference to follow is the *Reporting of Computational Modeling Studies in Medical Device Submissions* FDA guidance document [18]. Examples from the literature can be found in e.g., [19] and [20].
- **Derating.** The simplest approach to calculating exposure parameters is to derate the pressure by applying acoustic attenuation coefficients to the free field parameters. Skull and frequency-specific acoustic attenuation coefficients can be included to give an estimate of attenuation by the skull under different conditions [21].
  If using attenuation factors to calculate the *in situ* exposure parameters, the following should be reported:
  - The individual acoustic attenuation coefficients in dB.(MHz.cm)^−1^ or dB.cm^−1^ (for example, for skull, scalp and brain), and the values used for frequency and distance.
  - The total attenuation applied in dB.
  If experimental measurements are used to determine derating factors, for example using a sample of human skulls, the methods, assumptions, and variability should be reported [3].
- **Measuring the effect of the intracranial ultrasound field.** In some cases, indirect measurement of the physical effects of the ultrasound field on the brain can be made. For example, a temperature rise induced by the absorption of ultrasound energy could be measured using MR thermometry. Similarly, for some beam shapes, the bulk displacement induced by an acoustic radiation force could be measured using MR-ARFI. With some restrictions, these measurements could be correlated with the ultrasound exposure parameters in the brain [22].
  If using measurements to calculate the exposure parameters, the following should be reported:
  - Details of the measured quantity and the measurement method.
  - Details of how the exposure parameters are calculated from the measured quantity.

In principle, all of the spatial acoustic parameters described in Section 2 could be reported after accounting for the skull and brain. In particular, if using simulations to calculate exposure parameters, it is possible to extract the focal characteristics as well as pressure values at the spatial-peak and target locations [23]. As a minimum we recommend the following parameters are reported: (1) the *in situ* estimate for the spatial-peak pressure amplitude and its location, and (2) the *in situ* estimate for the pressure amplitude at the target, if it is known and different from the spatial-peak pressure amplitude. The former values are related to safety, the latter values are related to the study efficacy.

### 4.2 Estimate of *In Situ* Mechanical Index

For diagnostic ultrasound imaging, the mechanical index (MI) provides a standardised indicator related to the potential for mechanical bioeffects, specifically cavitation. In lieu of a safety standard specifically related to TUS, MI is also often reported in TUS studies in relation to regulatory limits given in the FDA guidance document [24]. A detailed discussion can be found in [25]. The *in situ* estimate of mechanical index is easily derived from the *in situ* estimate of spatial-peak pressure.

The mechanical index is formally defined as

(2) $$MI=\frac{p_{r,.3}}{\sqrt{f_{0}}},$$

where $p_{r,.3}$ is the peak-rarefactional pressure in MPa derated using an attenuation coefficient of 0.3 dB cm^−1^ MHz^−1^, and $f_{0}$ is the operating frequency in MHz (note units). This index and derating factor is intended to apply to ultrasound propagating in soft tissue. Note, in this expression, $p_{r}$ is used, as this is the formal definition of MI. Assuming operation in the linear regime, the peak positive and negative pressures are equal, and equal to the pressure amplitude, i.e., $p_{r}=p$, as used in elsewhere in this guide.

In TUS applications where ultrasound propagates through the skull, a more suitable derating factor may be used to define an alternative index, specific to this application, called the ${MI}_{tc}$, where the subscript “tc” refers to transcranial applications:

(3) $$MI_{tc}=\frac{p_{r,\alpha }}{\sqrt{f_{0}}}.$$

In this case, the method used to calculate the *in situ* or derated peak-rarefactional pressure $p_{r,\alpha }$ (for example, a simulation) should be reported. If derating a free field value, α, the attenuation coefficient, insertion loss of the skull, and any methods and parameters used for calculation should be reported. If modeling indicates that the target pressure is lower than the peak pressure, then the MI for both the spatial-peak and the target should be given.

### 4.3 Thermal Metrics

At least one of the following metrics should be reported.

- **Temperature Rise** In some cases, it may be possible to directly measure the temperature rise in soft tissue due to the applied TUS (for example, using MR thermometry or thermocouples), or to estimate the temperature rise in soft tissue using a thermal simulation. If reporting the temperature rise in soft tissue, the measurement or simulation method should be described as discussed in Sec. 4.1.
- **Thermal Index** For diagnostic ultrasound imaging, the thermal index (TI) provides a standardised indicator related to the potential for thermal bioeffects. TI is a unitless quantity intended to indicate a potential temperature rise in degrees celsius, but doesn’t directly correlate to tissue heating.^1^ In lieu of a safety standard specifically related to TUS, it is also often reported in TUS studies in relation to limits given in the FDA guidance document [24] as evidence of safety. Detailed discussions of these parameters can be found in [25]. Following IEC 62359 [25], the relevant thermal index is the bone-at-surface or cranial TI(TIC), which is calculated as
(4) $$TIC=\frac{W}{40D}.$$
  Here, W is the time-averaged acoustic power of the transducer in free field in mW, and D is the equivalent aperture diameter of the transducer in cm. For transducers in direct contact with the scalp, the equivalent aperture diameter would be taken to be the nominal aperture diameter of the transducer. However, when TUS transducers are applied with some stand off (such as a coupling pad) between the transducer surface and the scalp, the equivalent aperture diameter should be taken as the beam diameter at the scalp. This can be estimated from the distance between the transducer surface and the scalp, and the geometry of the transducer. Therefore, the transducer to scalp distance should be reported. If reporting TIC, the values for W and D (and the methods used to measure or calculate them) should also be reported.
- **Thermal Dose** The extent of biological changes in tissue resulting from thermal exposure is correlated with the amount of energy absorbed in tissue. However, for thermal energy, it is the temperature to which the tissue is raised, and the duration of the heating that plays the predominant biological role. Sapareto and Dewey [27] defined a ‘thermal isoeffective dose’ in terms of cumulative equivalent minutes (CEM) at 43°***C*** which allows conversion of any temperature-time (T-t) combination to the equivalent time for which the reference temperature of 43°***C*** must be applied to obtain the same level of thermal damage, given by
(5) $$CEM=\int_{t=0}^{t=final}\;R^{\left(43-T\right)}dt,$$
  where R=0.5 for T≥43° and R=0.25 for T<43°. This formula accounts for tissue thermotolerance (e.g. mediated by heat-shock proteins) which occurs during exposure at mild hyperthermic temperatures.

### 4.4 Spatial-Peak Pulse-Average Intensity

Acoustic intensity is a measure of the flow of energy from one point in an acoustic medium to another. The instantaneous acoustic intensity is a vector quantity (it depends on direction) and is given by the product of the acoustic pressure and acoustic particle velocity. For a plane wave, the pressure and particle velocity are related by the characteristic acoustic impedance of the medium (the product of sound speed and density). While plane wave expressions have only limited validity and cannot generally be applied throughout a focused acoustic field [7], they are often used to calculate the spatial-peak intensity in a focused field, where the wave is approximately plane.

Under this assumption, the instantaneous intensity in the direction of the plane wave can be calculated by

(6) $$I_{sp}(t)=\frac{p_{sp}{\left(t\right)}^{2}}{Z}.$$

Here, $p_{sp}(t)$ is the time varying acoustic pressure at the location of the spatial-peak, and Z is the characteristic acoustic impedance of the medium, which is approximately 1*.*5×10^6^ Rayls for soft tissue. For a harmonically-varying acoustic wave, assuming that the pulse duration is an integer multiple of the acoustic period (or otherwise long compared to the acoustic period), the intensity time-averaged over the pulse duration (generally called the pulse average intensity) can be calculated using

(7) $$I_{\text{sppa}}=\frac{1}{PDZ}\int_{0}^{PD}\;p_{sp}{\left(t\right)}^{2}dt,$$

which, for long pulses without ramping, reduces to

(8) $$I_{\text{sppa}}=\frac{p_{sp}^{2}}{2Z},$$

where $p_{sp}$ is the spatial-peak pressure amplitude (the amplitude of the sinusoidal pressure signal at the location of the spatial-peak). The $I_{sppa}$ should be easily derivable by the reader from the estimated *in situ* pressure and timing, and is therefore an optional reporting parameter. If reporting intensity values, the characteristic acoustic impedance Z used for the calculation must be reported.

### 4.5 Spatial-Peak Time-Average Intensities

The intensity time-averaged over the pulse train or pulse train repetition interval (generally called the time average intensity) can be calculated by

(9) $$I_{\text{spta}}=\frac{1}{TZ}\int_{0}^{T}\;p_{sp}{\left(t\right)}^{2}dt,$$

where T is the time period over which the average is taken. In the case of a pulse train without ramping, this reduces to

(10) $$I_{\text{spta,}\;\text{pulse}\;\text{train}}=DC_{\text{pulse}\;\text{train}}I_{\text{sppa}}.$$

Intensity parameters are widely reported in TUS studies. However, as mentioned above, it is important to note that in general, acoustic intensity is a vector quantity. Thus, intensity values calculated in this manner should always be reported alongside (not instead of) acoustic pressure values.

The pulse timing parameters used in TUS frequently include intermittent pulsing, often with different repetition intervals at different pulse levels (see Sec. 3). This causes ambiguity in the definition of time-averaged intensity, specifically regarding which time period should be used for averaging. Clearly, if 5 second pulse trains are administered every hour, taking the time-average over the latter period does not give a meaningful quantity.

Ultimately, the maximum continuous length of time where no TUS is administered included in the $I_{spta}$ calculation should be determined based on knowledge of characteristic diffusion times relevant to TUS. However, this is still a topic of active research, thus a recommendation cannot yet be made. For standardised reporting, our current recommendation is therefore to report the time period over which the average is taken. This can be done succinctly using the pulse level nomenclature introduced in Sec. 3, e.g. $I_{spta,\;\;pulse\;train}$ and $I_{spta,\;pulse\;train\;repeat}$.

### 4.6 Neuromodulation Dose Parameters

For TUS, the precise mechanisms through which ultrasound affects brain function are only just beginning to be understood. This means it is not yet possible to define or adopt appropriate dose parameters [28].

## 5 Summary

In this paper, we have suggested a minimal set of reporting guidelines for TUS parameters. In the supplementary material, we provide more details on how to obtain free field measurements. We provide some examples of how to estimate *In Situ* Parameters. We briefly list other aspects of the TUS experiment that fall outside of the scope of this paper but are also important in fully describing a study. We also provide a checklist of parameters to be reported.

## Supplementary Material

Supplement 1

## Figures and Tables

**Table 1.1:** Transducer and drive system description, drive system settings, and free-field pressure parameters adapted from Johnstone et al [5] and Badran et al. [6].

A						
Transducer and Drive System Parameters						
	Manufacturer, Model Number	Centre Frequency	Radius of Curvature	Aperture Diameter	Number Elements	Element Distribution
	
Transducer	TX1, Manufacturer 1	250 kHz	64 mm	64 mm	2	Spherical cap, annular array, equal area
Matching	Electrical impedance matching network, Manufacturer 1					
Integrated drive system	2-channel drive system, Manufacturer 1					

Drive System Settings			
	Operating Frequency	Output level Setting	Focal Position Setting
	
Experiment 1–4	270 kHz	4.78 W	Phases: 0, 66.3º

Free Field Pressure Parameters							
	Spatial Peak Pressure Amplitude	Axial Position Spatial Peak Pressure	Position of Centre of Axial -3dB Pressure	Axial -3dB Size	Lateral -3dB Size	Axial -6dB Size	Lateral -6dB Size
	
Experiment 1–4	700 kPa	43 mm	48 mm	30 mm	5 mm	48.7 mm	7 mm

B						
Transducer and Drive System Parameters						
	Manufacturer, Model Number	Centre Frequency	Radius of Curvature	Aperture Diameter	Number Elements	Element Distribution
	
Transducer	TX2, Manufacturer 2	650 kHz	80 mm	61 mm	1	Spherical cap
Integrated drive system	Ultrasound driving systems, Manufacturer 2					

Drive System Settings			
	Operating Frequency	Output level Setting	Focal Position Setting
	
Experiment 1	650 kHz	*pr* = 0.72 MPa	fixed

Free Field Pressure Parameters							
	Spatial Peak Pressure Amplitude	Axial Position Spatial Peak Pressure	Position of Centre of Axial -3dB Pressure	Axial -3dB Size	Lateral -3dB Size	Axial -6dB Size	Lateral -6dB Size
	
Experiment 1	720 kPa		80 mm			30 mm	5 mm

Notes: Reference plane is coincident with the centre of the radiating surface

Notes: Reference plane is coincident with the outer surface of the transducer housing

**Table 2.1:** Pulse timing parameters from two experiments in Johnstone *et al*. [5]. The A stimulation is experiment 1, condition A2 and the B stimulation is experiment 4, condition A2. In both cases, the first line contains the pulse characteristics and the second line contains the pulse train characteristics.

	Duration	Ramp Duration	Ramp Shape	Repetition Interval/ Frequency	Notes

A Pulse	2 s	0	rectangular	4 ms/250 Hz	
Pulse Train	0.3 s	0	rectangular		

B Pulse	3.25 ms	1 ms	Tukey on Pressure	4 ms/250 Hz	A 1s 250Hz square wave auditory mask was delivered using cicumaural headphones. The mask was synchronised to start 100 ms before the onset of TUS pulse trains.
Pulse Train	0.3 s	0	rectangular		

**Table 2.2.** Pulse timing parameters for the two stimulations from Zeng *et al*. [12]. A was a single pulse train repeated every 200 ms for 80 s. B consisted of a short pulse train repeated every 1.6 s, again for a total time of 80 s. This is an example of patterned repetitive TUS.

	Duration	Ramp Duration	Ramp Shape	Repetition Interval/Frequency	Notes

A Pulse	20 ms	0	rectangular	200 ms/5 Hz	
Pulse Train	80 ms	0	rectangular		

B Pulse	0.32 ms	0	rectangular	1 ms/1 kHz	
Pulse Train	500 ms	0	rectangular	1.6 s/0.625 Hz	
Pulse Train Repeat	80 s	0	rectangular		

**Table 2.3.** Pulse timing parameters from Gaur *et al*. [13] and Mohammadjavadi *et al*. [14]. Pulse trains had two levels of repetitions.

	Duration	Ramp Duration	Ramp Shape	Repetition Interval/Frequency	Notes

Pulse	0.5 ms	0	rectangular	1 ms/1 kHz	
Pulse Train	0.3 s	0	rectangular	1 s/1 Hz	
Pulse Train Repeat	30 s	0	rectangular	60 s/0.017 Hz	TUS and Light+TUS conditions were interleaved with Light Only and No Stimulus conditions
Repeat 2	40 min	0	rectangular		

## Footnotes

1 From 60601–2-37 [26]: “The TI gives a relative indication of the potential for temperature increase at a specific point along the ultrasound beam. The reason for the term “relative” is that the assumed conditions for heating in tissue are complex such that any single index or model cannot be expected to give the actual increase in temperature for all possible conditions and tissue types. Thus, for a particular beamshape, a TI of 2 represents a higher temperature rise than a TI of 1, but does not necessarily represent a rise of 2°*C*.”
